# Psychotherapeutische Sprechstunde am Arbeitsplatz: Zusammenhänge zwischen Unternehmensgröße und psychosomatischer Gesundheit

**DOI:** 10.1007/s00103-024-03904-7

**Published:** 2024-06-19

**Authors:** Nicole R. Hander, Julia Krohn, Fiona Kohl, Meike Heming, Yesim Erim, Regina Herold, Christoph Kröger, Marieke Hansmann, Volker Köllner, Sophia Chrysanthou, Uta Wegewitz, Ute B. Schröder, Manuel Feißt, Kristin Herrmann, Eva Rothermund

**Affiliations:** 1 Klinik für Psychosomatische Medizin und Psychotherapie, Universitätsklinikum Ulm, Albert-Einstein-Alle 23, 89081 Ulm, Baden-Württemberg Deutschland; 2 Institut für Arbeits‑, Sozial- und Umweltmedizin, Centre for Health and Society, Medizinische Fakultät und Universitätsklinikum Düsseldorf, Heinrich-Heinrich-Universität Düsseldorf, Düsseldorf, Nordrhein-Westfalen Deutschland; 3 Psychosomatische und Psychotherapeutische Abteilung, Universitätsklinikum Erlangen, Friedrich-Alexander-Universität Erlangen-Nürnberg (FAU), Erlangen, Bayern Deutschland; 4 Abteilung Klinische Psychologie und Psychotherapie, Institut für Psychologie, Universität Hildesheim, Hildesheim, Niedersachsen Deutschland; 5 Medizinische Klinik mit Schwerpunkt Psychosomatik, Forschungsgruppe Psychosomatische Rehabilitation, Charité Universitätsmedizin Berlin, Berlin, Berlin Deutschland; 6 FG 3.5 Evidenzbasierte Arbeitsmedizin, Betriebliches Gesundheitsmanagement, b a u a: Bundesanstalt für Arbeitsschutz und Arbeitsmedizin, Berlin, Berlin Deutschland; 7 Institut für Medizinische Biometrie, Universitätsklinikum Heidelberg, Heidelberg, Baden-Württemberg Deutschland

**Keywords:** Betriebliche Gesundheitsförderung, KMU, Psychische Gesundheit, Früherkennung and Prävention, Psychosoziales Sicherheitsklima, Workplace health promotion, Mental health, SME, Early diagnosis and prevention, Psychosocial safety climate

## Abstract

**Hintergrund:**

Die Inanspruchnahme der psychotherapeutischen Sprechstunde am Arbeitsplatz (PT-A) wurde bislang in Großunternehmen (GU) untersucht. Diese unterscheiden sich strukturell von mittleren und Klein(st)unternehmen (KMU). Unterschiede der Nutzerprofile einer PT‑A hinsichtlich psychosomatischer Gesundheit, arbeitsbezogener Selbstwirksamkeit und Arbeitsfähigkeit sowie des psychosozialen Sicherheitsklimas (PSC) abhängig von der Unternehmensgröße wurden bisher kaum betrachtet.

**Methoden:**

In der Interventionsstudie „Frühe Intervention am Arbeitsplatz“ (friaa) wurden zwischen 09/2021 und 01/2023 an einer PT‑A interessierte Beschäftigte aus GU und KMU deutschlandweit befragt. Mittels t‑ und χ^2^-Tests wurden Unterschiede zwischen Beschäftigten in GU (*n* = 439) und KMU (*n* = 109) hinsichtlich F‑Diagnosen nach ICD-10 („International Statistical Classification of Diseases and Related Health Problems“; psychische und Verhaltensstörungen), Depressivität (PHQ-9), Ängstlichkeit (GAD-2), allgemeinen Funktionsniveaus (GAF), somatischer Symptombelastung (SSS-8), Gesundheit (VR-12), Arbeitsfähigkeit (WAI), Selbstwirksamkeit (SOSES) und psychosozialen Sicherheitsklimas (PSC-4) geprüft und mittels Korrelationsanalyse explorativ deren Zusammenhänge untersucht.

**Ergebnisse:**

Beide Gruppen zeigten sich ähnlich stark beansprucht. In GU wurden aus Sicht der Beschäftigten psychosoziale Themen signifikant häufiger thematisiert als in KMU mit einer mittleren Effektgröße. Die Studie lieferte erste Hinweise, dass in GU positive Zusammenhänge des PSC‑4 mit SOSES und WAI sowie negative mit PHQ‑9 und SSS‑8 vorliegen.

**Diskussion:**

Die vergleichbare psychische Beanspruchung der Beschäftigten in GU und KMU weist auf den Bedarf von verhältnis- und verhaltenspräventiven Maßnahmen unabhängig von der Unternehmensgröße hin. Vor allem in KMU sollte die Thematisierung psychosozialer Gesundheit einen größeren Stellenwert einnehmen.

## Abstract (en)

**Background:**

The utilization of psychotherapeutic consultation at work (PT-A) has so far been investigated in large enterprises (LEs). These differ structurally from small(est) and medium-sized enterprises (SMEs). Differences in the user profiles of a PT‑A with regard to psychosomatic health, work-related self-efficacy, and work ability depending on company size have hardly been investigated. This study also examined differences in the employees’ perception of the psychosocial safety climate (PSC) in the company, which represents management’s efforts to promote mental health.

**Methods:**

As part of the Early Intervention in the Workplace intervention study called “friaa”, employees from LEs and SMEs interested in a PT‑A were surveyed throughout Germany from September 2021 to January 2023. Using t‑ and χ^2^-tests, differences between employees in LEs (*n* = 439) and SMEs (*n* = 109) were examined with regard to the ICD-10 F diagnostic code (“International Statistical Classification of Diseases and Related Health Problems”; mental and behavioral disorders), depression (PHQ-9), anxiety (GAD-2), level of functioning (GAF), somatic symptom burden (SSS-8), health (VR-12), ability to work (WAI), self-efficacy (SOSES), and psychosocial safety climate (PSC-4). The association between these variables and especially the PSC‑4 were investigated using correlation analysis.

**Results:**

Both groups showed similar levels of stress. From the employees’ perspective, psychosocial issues were addressed significantly more frequently in LEs than in SMEs with a medium effect size. The study provided initial indications that in LEs there were positive correlations of the PSC‑4 with SOSES and WAI and negative ones with PHQ‑9 and SSS‑8.

**Discussion:**

The comparable psychological strain on employees in LEs and SMEs points to the need for behavioral and structural preventive measures regardless of the company size. Mainly in SMEs, organizational communication of psychosocial health should be given greater priority.

## Hintergrund

Die Anzahl von Arbeitsunfähigkeitstagen aufgrund von psychischen Erkrankungen ist zwischen 2011 und 2019 um 42 % und zwischen 2011 und 2021 um 53,2 % gestiegen [1]. Die 2 häufigsten Diagnosen stellen Depressionen und Anpassungsstörungen dar [2]. Mit 42 % sind psychische und Verhaltensstörungen die Hauptursache für eine Erwerbsminderungsrente [3]. Der Arbeitsplatz und die psychische Gesundheit stehen dabei in einem engen Zusammenhang [4]. Die Ergebnisse eines systematischen Reviews [5] zeigen verschiedene Risikofaktoren am Arbeitsplatz, wohingegen die Größenklasse der Unternehmen als Einflussfaktor bisher kaum Berücksichtigung fand. Die psychotherapeutische Sprechstunde am Arbeitsplatz zeichnet sich durch einen niederschwelligen Erstkontakt, Beratung in allen Phasen der psychischen Erkrankung, Arbeitsplatzbezug und Vernetzung mit Mitbehandelnden aus [6]. Ersten Studien in Großunternehmen (GU) zufolge scheint sie eine hilfreiche Intervention zu sein, um Beschäftigte früh im Erkrankungsverlauf zu erreichen [7, 8] und die Arbeitsunfähigkeitstage zu reduzieren [9].

In Deutschland waren im Jahr 2021 ca. 21,5 Mio. Personen in Klein(st)- und mittleren Unternehmen (KMU) und 16,9 Mio. in GU beschäftigt.^1^ Untersucht wurden bereits charakteristische Merkmale der Unternehmensgröße [10]. In GU, welche sich entsprechend der Europäischen Kommission durch mehr als 249 Beschäftigte^2^ definieren, liegen häufig bessere Karriereperspektiven und eine höhere Jobsicherheit im Gegensatz zu KMU vor [11]. Die organisatorischen Strukturen sind tendenziell hierarchischer, unflexibler und formeller ausgestaltet. Beschäftigte leiden häufig unter qualitativer Arbeitsüberlastung [12]. Im Vergleich dazu ist die Arbeit in KMU oft flexibler und autonomer gestaltbar, mit tendenziell guten zwischenmenschlichen Arbeitsbeziehungen [11], jedoch mit einer häufig hohen quantitativen Arbeitslast [12].

Zum Schutz der Beschäftigten vor physischen und psychischen Schäden wurde 1989 die Europäische Rahmenrichtlinie 89/391/EWG eingeführt. Arbeitgebende sind bspw. dazu verpflichtet, regelmäßige Gefährdungsbeurteilungen psychischer Belastung nach § 5 ArbSchG [13] zu erheben. In Deutschland wurde diese von 57 % der Kleinst- und 29 % der Kleinunternehmen nicht umgesetzt, wohingegen 70 % der GU die Gefährdungsbeurteilung implementierten [14]. Ursachen für die geringe Implementierung in KMU sind oft mangelnde finanzielle und personelle Ressourcen [11]. Zudem fehlt es Arbeitgebenden an Wissen und Aufklärung über die Langzeitfolgen von psychischen Störungen [11], obwohl diese von höchster Relevanz sind, da sie mit einer verringerten Produktivität und erhöhten Fehlzeiten [15] zusammenhängen. Daher sollten Arbeitgebende auch aus Wirtschaftlichkeit Interesse an der psychosomatischen Gesundheit der Beschäftigten haben [16, 17]. Arbeitgebende nehmen selbst eine wichtige Funktion in der Wahrung des psychischen Wohlbefindens ein: Über die Einschätzung des psychosozialen Sicherheitsklimas („Psychosocial Safety Climate“, PSC) lässt sich die Bereitschaft des Managements, im Sinne der psychischen Gesundheit der Beschäftigten zu handeln, messen [17]. Doch ob Unterschiede in der Wahrnehmung des PSC von Beschäftigten aus KMU und GU vorliegen, bleibt zu klären.

Der Einfluss der Unternehmensgröße und des kontextabhängigen betrieblichen Gesundheitsmanagements (BGM; [18]) auf die Gesundheit der Beschäftigten wurde bislang kaum betrachtet [11]. Erste Studien [19], die Kleinst- und größere Unternehmen verglichen haben, stellten fest, dass das Risiko für depressive oder Angsterkrankungen in Kleinstunternehmen (bis 9 Beschäftigte) am geringsten ausgeprägt war. Dieses Ergebnis steht im Einklang mit einer Querschnittserhebung aus dem Vereinigten Königsreich (UK; [20]), die darauf hindeutete, dass Beschäftigte in KMU eine geringere Stressbelastung erlebten als in GU.

Studien zur Evaluation der PT‑A, die bisher lediglich in GU durchgeführt wurden, zeigten, dass die Nutzergruppe der PT‑A weniger beansprucht war als bspw. jene in der ambulanten Regelversorgung [7, 8, 21]. Aufgrund ungleicher Strukturen (z. B. stärker implementierte BGM-Strukturen in GU als in KMU; [18, 22]) kann vermutet werden, dass Beschäftigte in Abhängigkeit von der Unternehmensgröße zu verschiedenen Zeitpunkten der psychischen Erkrankung durch die PT‑A erreicht werden. Da es in Deutschland mehr Beschäftigte in KMU als in GU gibt,^1^ ist es wichtig, die Nutzergruppe der vielversprechenden PT‑A nicht nur in GU, sondern kontextabhängig auch in KMU zu untersuchen.

Ziel dieser Arbeit war es, explorativ zu untersuchen, ob sich Beschäftigte, die sich in einer PT‑A vorstellten, je nach Unternehmensgröße (GU oder KMU) hinsichtlich ihrer psychosomatischen Gesundheit, ihres allgemeinen Funktionsniveaus, ihrer Arbeitsfähigkeit und berufsbezogenen Selbstwirksamkeit unterscheiden. Zudem wurden die Zugangswege zur PT‑A verglichen. Auch wurden Unterschiede im Hinblick auf die Dimensionen des PSC betrachtet sowie dessen Zusammenhänge mit dem allgemeinen Funktionsniveau, selbstberichteter Depressivität, Ängstlichkeit, somatischer Symptombelastung, Arbeitsfähigkeit und berufsbezogener Selbstwirksamkeit explorativ untersucht.

## Methoden

### Studiendesign und -population

Die Daten wurden in der randomisiert-kontrollierten Interventionsstudie „Frühe Intervention am Arbeitsplatz“ (friaa) erhoben [23], welche den Nutzen einer PT‑A im Vergleich zur Treatment-as-usual-Behandlung bei psychisch beanspruchten Beschäftigten evaluiert. Die Teilnehmenden wurden in den 5 Studienzentren Ulm, Düsseldorf, Erlangen, Hildesheim und Teltow von 09/2021 bis 01/2023 in Zusammenarbeit mit betriebsärztlichem Fachpersonal und Führungskräften der teilnehmenden Unternehmen rekrutiert. Zusätzlich wurden Beschäftigte über Zeitungen und soziale Medien angesprochen. Mittels Selbstbeurteilungsbögen wurden Teilnehmende zu arbeits- und gesundheitsbezogenen Themen befragt und F‑Diagnosen durch klinische Interviews erhoben. Eingeschlossen wurden Beschäftigte zwischen 18 und 65 Jahren mit ausreichenden Deutschkenntnissen und einem Beschäftigungsverhältnis von mindestens 15 Wochenstunden. Zudem mussten diese entweder eine den ICD-10-Kriterien [24] entsprechende F‑Diagnose (Common Mental Disorder) oder eine allgemeine Funktionseinschränkung, erfasst mittels der GAF-Skala (Global Assessment of Functioning Scale) mit einem Wert <81 [25], aufweisen. Ausschlusskriterien waren eine laufende Psychotherapie oder ein laufender Antrag auf (Früh‑)Berentung. Die Daten entspringen der klinischen Ersterhebung vor der Interventions- oder Kontrollbedingung, wobei die Zuteilung zur Bedingung nicht berücksichtigt wurde [23].

### Variablen

Die Einteilung der Beschäftigten erfolgte abhängig von der Unternehmensgröße: Mittlere und Klein(st)unternehmen (KMU) wurden „als Unternehmen mit weniger als 250 Beschäftigten“ definiert.^2^

Zur soziodemografischen und arbeitsplatzbezogenen Beschreibung der Stichprobe wurden mitunter Geschlecht, Alter, Partnerschaft, Wochenarbeitsstunden, Berufsgruppe, Führungsposition, Erwerbssituation und Schichtdienst erfragt. Zudem wurden Zugangswege zur PT‑A erhoben.

Es wurden Variablen zur Erfassung der psychischen Gesundheit, der Arbeitsfähigkeit und des PSC erhoben:

Das Depressionsmodul („Patient-Health-Questionnaire 9“, PHQ-9) des „Gesundheitsfragebogens für Patienten“ (PHQ-D) ist ein valides und reliables Instrument zur Erfassung von Depressivität mittels 9 Items [26, 27]. Auf einer 4‑stufigen Likert-Skala wurde die Zustimmung zu Items wie „wenig Interesse oder Freude an Ihren Tätigkeiten“ erfragt. Je höher der Gesamtsummenscore (Range: 0–27), desto höher die Belastung; ab einem Wert von 10 handelt es sich um eine moderate und ab 15 um eine mittelgradige depressive Symptomatik [28].

Der GAD‑2 („Generalized Anxiety Disorder 2“) ist ein verkürzter Fragebogen des GAD‑7 mit ähnlich guter Reliabilität und Validität zur Erfassung von Angststörungen mithilfe von 2 Items [29]. Beschwerden wie Nervosität und Gedankenkreisen innerhalb der letzten 2 Wochen wurden mittels einer 4‑stufigen Skala erfragt. Je höher der Gesamtsummenscore (Range: 0–6), desto höher die beschwerdebedingte Belastung. Ein Score von ≥3 ist ein Indikator für die pathologische Ausprägung einer Angststörung [29].

Es wurde mittels des klinischen Interviews „Mini-International Neuropsychiatric Interview“ (M.I.N.I.; [30]) geprüft, ob sich die Beschwerden der Teilnehmenden durch eine F‑Diagnose nach ICD-10 (International Statistical Classification of Diseases and Related Health Problems; [24]) einordnen lassen.

Anhand der GAF-Skala (0–100 Punkte) wurde der Schweregrad der Symptome oder das allgemeine Funktionsniveau der Beschäftigten in den psychischen, sozialen und beruflichen Bereichen eingeschätzt. Je höher der Wert, desto besser die Leistungsfähigkeit [25].

Mittels der „Somatic Symptom Scale 8“ (SSS-8) wurden reliabel und valide somatische Symptome erhoben. Die Beeinträchtigung durch Symptome, wie bspw. Bauchschmerzen oder Rückenschmerzen, wurde auf einer 4‑stufigen Skala gemessen, wobei ein höherer Gesamtsummenscore eine höhere Belastung bedeutet (Range: 0–32). Ab einem Gesamtsummenscore von 8 spricht man von mittlerer, ab 12 von hoher und ab 16 von sehr hoher Belastung [31].

Mittels eines Items des „Veterans RAND 12-Item Health Survey“ (VR-12) wurde auf einer Skala von 1 = „ausgezeichnet“ bis 5 = „schlecht“ der allgemeine Gesundheitsstatus erhoben [32].

Der „Work Ability Index“ (WAI) umfasst 10 Fragen zur reliablen und validen Erfassung der Arbeitsfähigkeit. Es wurde das erste Item verwendet, mit welchem die derzeitige Arbeitsfähigkeit im Vergleich zur besten, je erreichten Arbeitsfähigkeit auf einer Skala von 0 bis 9 erfasst wird [33].

Die „Short Occupational Self Efficacy Scale“ (SOSES) ist ein reliables und valides Messinstrument bestehend aus 6 Items (z. B. „Ich fühle mich den meisten beruflichen Anforderungen gewachsen“) zur Erfassung der arbeitsbezogenen Selbstwirksamkeit auf einer Skala von 1 bis 6. Je höher der Mittelwert der Skala, desto höher die arbeitsbezogene Selbstwirksamkeit [34].

Das psychosoziale Sicherheitsklima („Psychosocial Safety Climate“, PSC) beschreibt aus der Perspektive der Beschäftigten die Bereitschaft des Managements, im Sinne der psychischen Gesundheit der Beschäftigten zu handeln [17]. Die verwendete reliable und valide Kurzversion (PSC-4) besteht aus 4 Items, die jeweils eine Dimension erfassen (Engagement des Managements bei der Stressprävention, psychische Gesundheit als Priorität und genauso wichtig wie die Produktivität, Kommunikation psychosozialer Themen im Unternehmen, Partizipation der Beschäftigten bei der Stressprävention) und mittels einer 5‑stufigen Likert-Skala von 1 bis 5 beantwortet werden [35].

### Statistische Analysen

Mittels t‑Tests wurden die Mittelwertsunterschiede der stetigen Variablen und mittels Χ^2^-Tests die Unterschiede der kategorialen Variablen zwischen Beschäftigten aus GU und KMU überprüft. Die Effektgröße wurde bei t‑Tests mittels Cohens d und bei Χ^2^-Tests mittels Cramers V bestimmt [36]: kleiner Effekt |d| ≤ 0,2 und V = 0,1, mittlerer Effekt |d| = 0,5 und V = 0,3, großer Effekt |d| = 0,8 und V = 0,5. Bei der Analyse der Mittelwertsunterschiede wurde zusätzlich für die Variablen Geschlecht, Alter, Wochenarbeitsstunden, Führungsposition und Schichtdienst mittels einer einfaktoriellen Kovarianzanalyse („analysis of covariance“ – ANCOVA) statistisch kontrolliert und die Effektstärke mittels partiellen Eta-Quadrats (η_p_^2^) dargestellt. In Übereinstimmung mit Cohen [36] wurde η_p_^2^ = 0,01 als kleiner, η_p_^2^ = 0,03 als mittlerer und η_p_^2^ = 0,14 als großer Effekt betrachtet. Zur Berechnung von Zusammenhängen zwischen den Dimensionen des PSC‑4 und den stetigen Variablen wurde eine Korrelationsanalyse mittels Spearman-Korrelationskoeffizienten getrennt für KMU und GU durchgeführt, wobei r ≤ 0,1 als kleiner Effekt, 0,1 < r ≤ 0,3 als mittlerer Effekt und r ≥ 0,5 als großer Effekt interpretiert wurde [37]. Die interne Konsistenz der Skalen wurde mithilfe von Cronbachs Alpha geschätzt. Das Signifikanzniveau wurde auf 5 % (*p* = 0,05) gesetzt. Aufgrund multipler Testung derselben zugrunde liegenden Stichprobe wurden signifikante Ergebnisse der t‑ und Χ^2^-Tests zusätzlich nach Bonferroni-Holm korrigiert. Alle Analysen erfolgten mit der SPSS Version 29.

## Ergebnisse

### Stichprobenbeschreibung

Es nahmen 60 Betriebe teil. Zwischen September 2021 und Januar 2023 stellten sich 679 Beschäftigte in der PT‑A vor, von welchen 550 eingeschlossen werden konnten. Davon stammten *n* = 439 aus GU und *n* = 109 aus KMU; bei *n* = 2 fehlten diesbezügliche Angaben. Der Zugang zur PT‑A (Tab. 1) erfolgte in KMU mehrheitlich eigeninitiativ (KMU = 64,2 %, GU = 40,5 %), gefolgt von „auf Empfehlung durch den Betriebsärztlichen Dienst“ (BÄD; KMU = 10,1 %, GU = 15,9 %) und „durch Angehörige“ (KMU = 10,1 %, GU = 3,9 %). In GU hingegen nahm neben dem BÄD auch der Sozialdienst eine wichtige Rolle ein (GU = 23,9 %, KMU = 6,4 %).

**Tab. 1:** Soziodemografische Merkmale von Teilnehmenden aus Großunternehmen (GU) und Klein(st)- und mittleren Unternehmen (KMU) sowie Zugangswege zur PT‑A

**–**	**GU (*n*** **=** **439)**	**KMU (*n*** **=** **109)**	**t (df)**	***p***
	**Mittelwert (SD)**	**Mittelwert (SD)**		
*Alter (Jahre)*	46,36 (10,91)	44,01 (11,47)	t (546) = 1,99	0,047
*Arbeitsstunden/Woche*	36,88 (7,00)	36,88 (7,72)	t (543) = −0,00	0,999
**–**	**% (*n*)**	**% (*n*)**	**χ**^**2**^** (df)**	***p***
*Geschlecht*				
Männlich	46,7 (205)	37,6 (41)	χ^*2*^ (2) = 3,23	0,199
Weiblich	53,1 (233)	62,4 (68)	–	–
Divers	0,2 (1)	0 (0)	–	–
*Partnerschaft*				
Verheiratet/in fester Beziehung	60,8 (267)	59,6 (65)	χ^*2*^ (3) = 2,01	0,570
Ledig	25,3 (111)	30,3 (33)	–	–
Geschieden	11,8 (52)	9,2 (10)	–	–
Verwitwet	2,1 (9)	0,9 (1)	–	–
*Berufsgruppe*				
Arbeiter/-in	23,9 (105)	17,4 (19)	χ^*2*^ (4) = 21,23	<0,001
Selbstständig	0 (0)	2,8 (3)	–	–
Angestellt	65,6 (288)	60,6 (66)	–	–
Beamter/Beamtin	5,0 (22)	6,4 (7)	–	–
Andere	5,5 (24)	12,8 (14)	–	–
*Führungsposition*				
Ja	20,5 (90)	23,9 (26)	χ^*2*^ (1) = 0,59	0,443
Nein	79,5 (349)	76,1 (83)	–	–
*Erwerbssituation*				
Vollzeit	75,2 (330)	65,1 (71)	χ^*2*^ (4) = 5,65	0,227
Teilzeit	23,5 (103)	32,1 (35)	–	–
Azubi	0,7 (3)	1,8 (2)	–	–
Freiwillig (unentgeltlich) beschäftigt	0,2 (1)	0 (0)	–	–
Geschützte Tätigkeit	0 (0)	0 (0)	–	–
Nicht zutreffend	0,5 (2)	0,9 (1)	–	–
*Schichtdienst*				
Ja	14,1 (62)	10,1 (11)	χ^*2*^ (1) = 1,23	0,268
Nein	85,9 (377)	89,9 (98)	–	–
*Zugangswege zur PT‑A*				
Betriebsrat	2,1 (9)	0,9 (1)	χ^*2*^ (9) = 46,88	<0,001
Betriebsärztlicher Dienst	15,9 (70)	10,1 (11)	–	–
Personalabteilung	1,1 (5)	4,6 (5)	–	–
Sozialdienst	23,9 (105)	6,4 (7)	–	–
BEM	3,2 (14)	0,9 (1)	–	–
Kolleginnen und Kollegen	3,2 (14)	0 (0)	–	–
Vorgesetzte	2,5 (11)	0 (0)	–	–
Eigeninitiative	40,5 (178)	64,2 (70)	–	–
Angehörige	3,9 (17)	10,1 (11)	–	–
Sonstige	3,6 (16)	2,8 (3)	–	–

*N* = 545–548

*SD* Standardabweichung; *Df* Freiheitsgrade; *PT‑A* Psychotherapeutische Sprechstunde am Arbeitsplatz

Die Teilnehmenden in GU waren durchschnittlich 46 Jahre alt (SD = 10,9), in KMU 44 Jahre (SD = 11,5). Die Mehrheit der Teilnehmenden war weiblich (GU = 53,1 %, KMU = 62,4 %), in einer festen Partnerschaft (GU = 60,8 %, KMU = 59,6 %), ohne Führungsposition (GU = 79,5 %, KMU = 76,1 %), ohne Schichtdienst (GU = 85,9 %, KMU = 89,9 %) und in einer Arbeitswoche von durchschnittlich 36,9 h beschäftigt (Tab. 1). Beschäftigte aus KMU und GU unterschieden sich nur in Bezug auf das Alter und die Berufsgruppe signifikant.

### Klinische Parameter

Es zeigten sich keine Unterschiede bezüglich der psychosomatischen Gesundheit anhand der Gesamtskalen zwischen der Nutzergruppe der PT‑A in GU und in KMU (Tab. 2). In GU und KMU lag der Gesamtsummenscore des PHQ‑9 (GU: M = 12,94, SD = 5,13; KMU: M = 12,7, SD = 5,03) und des GAD‑2 (GU: M = 3,46, SD = 1,72; KMU: M = 3,41, SD = 1,66) über dem Schwellenwert, womit Anzeichen für eine moderate depressive und ängstliche Symptomatik vorhanden waren. Die häufigsten Diagnosen waren depressive (GU: 51,5 %; KMU: 45,9 %) und Anpassungsstörungen (GU: 15,3 %; KMU: 18,3 %). Ohne F‑Diagnose und mit GAF <81 wurden in GU 14,1 % und in KMU 20,2 % vorstellig. Das Funktionsniveau lag im Bereich von 70–61 (GU: M = 66,27, SD = 7,76; KMU: M = 66,08, SD = 6,95) und umfasste „einige leichte Symptome …, aber im allgemeinen relativ gute Leistungsfähigkeit …“ [25]. Auch somatoforme Auswirkungen lagen bei Beschäftigten in GU und KMU vor (GU: M = 13,44, SD = 5,82; KMU: M = 12,77, SD = 5,57). Der generelle Gesundheitsstatus wurde als „weniger gut“ bis „gut“ beschrieben (GU: M = 3,49, SD = 0,71; KMU: M = 3,44, SD = 0,72) und die selbsteingeschätzte Arbeitsfähigkeit lag im mittleren arbeitsfähigen Bereich (GU: M = 5,05, SD = 2,38; KMU: M = 5,13, SD = 2,55). Die arbeitsbezogene Selbstwirksamkeit wurde tendenziell als gut bewertet (GU: M = 3,85 SD = 0,97; KMU: M = 3,94, SD = 1,07).

**Tab. 2:** Unterschiede zwischen Beschäftigten in Großunternehmen (GU) und Klein(st)- und mittleren Unternehmen (KMU) hinsichtlich ihrer psychosomatischen Gesundheit, allgemeinen Funktionsniveaus, Arbeitsfähigkeit, arbeitsbezogener Selbstwirksamkeit und Dimensionen des PSC

**–**	**GU (*n*** **=** **439)**	**KMU (*n*** **=** **109)**	**t (df)**	**Cohens d**	**ANCOVA**	**η**_**p**_^**2**^
	**Mittelwert (SD)**	**Mittelwert (SD)**			**F (df)**	
PHQ‑9^a,b^ gesamt	12,94 (5,13)	12,70 (5,03)	t (546) = 0,44; *p* = 0,663	0,05	F (1,538) = 0,28; *p* = 0,595	0,001
GAD‑2^c^ gesamt	3,46 (1,72)	3,41 (1,66)	t (546) = 0,28; *p* = 0,777	0,03	F (1,538) = 0,31; *p* = 0,581	0,001
GAF^d^	66,27 (7,76)	66,08 (6,95)	t (541) = 0,23; *p* = 0,816	0,03	F (1,533) = 0,36; *p* = 0,550	0,001
SSS‑8^e^ gesamt	13,44 (5,82)	12,77 (5,57)	t (533) = 1,07; *p* = 0,287	0,12	F (1,525) = 1,03; *p* = 0,311	0,002
VR^f^	3,49 (0,71)	3,44 (0,72)	t (545) = 0,59; *p* = 0,554	0,06	F (1,537) = 0,00; *p* = 0,981	0,000
WAI^g^	5,05 (2,38)	5,13 (2,55)	t (546) = −0,29; *p* = 0,775	−0,03	F (1,538) = 0,00; *p* = 0,970	0,000
SOSES^h^ gesamt	3,85 (0,97)	3,94 (1,07)	t (545) = −0,83; *p* = 0,410	−0,09	F (1,537) = 0,32; *p* = 0,569	0,001
PSC‑4^i^ Item 1: Engagement	2,52 (1,08)	2,54 (1,11)	t (542) = −0,12; *p* = 0,904	−0,01	F (1,534) = 0,01; *p* = 0,935	0,000
PSC‑4^i^ Item 2: Priorität	2,75 (1,15)	2,81 (1,21)	t (541) = −0,47; *p* = 0,642	−0,05	F (1,533) = 0,20; *p* = 0,655	0,000
PSC‑4^i^ Item 3: Kommunikation	2,67 (1,20)	2,17 (1,16)	t (543) = 3,95; *p* < 0,012*	0,43	F (1,535) = 16,28; *p* < 0,011*	0,030
PSC‑4^i^ Item 4: Partizipation	2,46 (1,10)	2,30 (0,99)	t (540) = 1,38; *p* = 0,169	0,15	F (1,532) = 2,81; *p* = 0,094	0,005
**–**	**% (*n*)**	**% (*n*)**	**χ**^**2**^** (df)**	**Cramers V**	**–**	**–**
*Diagnosen*^*j*^						
Depressive Störungen	51,5 (226)	45,9 (50)	χ^*2*^ (9) = 10,40; *p* = 0,319	0,14	–	–
Anpassungsstörungen	15,3 (67)	18,3 (20)	–	–	–	–
Keine psychische Primärdiagnose	14,1 (62)	20,2 (22)	–	–	–	–
Angststörungen	7,1 (31)	8,3 (9)	–	–	–	–
Somatoforme Störungen	3,6 (16)	2,8 (3)	–	–	–	–
Posttraumatische Belastungsstörungen	2,5 (11)	0,9 (1)	–	–	–	–
Manische/bipolare Störungen	0,9 (4)	2,8 (3)	–	–	–	–
Zwangsstörungen	0,2 (1)	0 (0)	–	–	–	–
Sonstige	4,1 (18)	0,9 (1)	–	–	–	–
Fehlende Angaben	0,7 (3)	0 (0)	–	–	–	–

Die signifikanten Werte des PSC-4: Item 3 *p* < 0,001 wurden anhand der Bonferroni-Holm-Korrektur adjustiert und adjustiert in der Tabelle vermerkt; in der ANCOVA mit Unternehmensgröße als Zwischensubjektfaktor wurde für die Variablen Alter, Geschlecht, Wochenarbeitsstunden, Schichtdienst und Führungsposition kontrolliert

*SD* Standardabweichung; *df* Freiheitsgrade

^a^[26]

^b^[27]

^c^[29]

^d^[25]

^e^[31]

^f^[32]

^g^[33]

^h^[34]

^i^[35]

^j^F‑Diagnose und GAF ≥81 *n* = 2 Personen

*p < 0,05; **p < 0,01

Die Dimensionen des psychosozialen Sicherheitsklimas wurden in beiden Gruppen im verbesserungswürdigen Bereich bewertet (Tab. 2). Die „Kommunikation psychosozialer Themen“ unterschied sich signifikant mit einer mittleren Effektgröße (t (543) = 3,95; *p* < 0,001): Beschäftigte berichteten von ihrem Betrieb, dass in GU psychosoziale Themen häufiger angesprochen wurden (M = 2,67; SD = 1,20) als in KMU (M = 2,17; SD = 1,16). Dieser Unterschied blieb nach der Bonferroni-Holm-Korrektur (*p* < 0,012) und der Kontrolle für Kovariablen bestehen (F (1,535) = 16,28; *p* < 0,001).

Bei explorativer Betrachtung der Unterschiede der Einzelitems zeigte sich, dass sich gemäß des SSS‑8 (t (544) = 2,07; *p* = 0,039) Beschäftigte in GU durch Schlafstörungen mehr beeinträchtigt (M = 2,48; SD = 1,32) fühlten als Beschäftigte in KMU (M = 2,18; SD = 1,31) bei kleiner Effektgröße. Das Item des PHQ‑9 zeigte eine ähnliche Tendenz (t (546) = 1,91; *p* = 0,056). Dieser Unterschied blieb nach der Bonferroni-Holm-Korrektur und der Kontrolle für Kovariablen nicht bestehen (Tab. 3).

**Tab. 3:** Unterschiede zwischen Beschäftigten in Großunternehmen (GU) und Klein(st)- und mittleren Unternehmen (KMU) hinsichtlich ihrer psychosomatischen Gesundheit und arbeitsbezogenen Selbstwirksamkeit bei Betrachtung der Einzelitems

	GU (*n* = 439)	KMU (*n* = 109)	t (df)	Cohens d	ANCOVA	η_p_^2^
	Mittelwert (SD)	Mittelwert (SD)			F (df)	
*PHQ‑9*^a,b^						
a: Interesselosigkeit oder Freudverlust	1,70 (0,86)	1,70 (0,96)	t (546) = 0,07; *p* = 0,944	0,01	F (1,538) = 0,00; *p* = 0,956	0,000
b: Niedergeschlagenheit	1,59 (0,93)	1,63 (0,86)	t (546) = −0,47; *p* = 0,642	−0,05	F (1,538) = 0,19; *p* = 0,665	0,000
c: Schlafstörungen	2,06 (1,02)	1,85 (0,95)	t (546) = 1,91; *p* = 0,056	0,21	F (1,538) = 3,26; *p* = 0,071	0,006
d: Müdigkeit oder energielos	2,21 (0,86)	2,11 (0,81)	t (546) = 1,07; *p* = 0,285	0,12	F (1,538) = 1,62; *p* = 0,204	0,003
e: Appetitveränderung (vermindert/erhöht)	1,45 (1,03)	1,41 (1,01)	t (546) = 0,33; *p* = 0,743	0,04	F (1,538) = 0,26; *p* = 0,610	0,000
f: Negatives Selbstbild	1,32 (1,06)	1.24 (1,01)	t (546) = 0,74; *p* = 0,463	0,08	F (1,538) = 0,91; *p* = 0,341	0,002
g: Konzentrationsstörungen	1,54 (0,95)	1,61 (0,96)	t (546) = −0,69; *p* = 0,492	−0,07	F (1,538) = 0,22; *p* = 0,640	0,000
h: Unruhig oder verlangsamt	0,70 (0,88)	0,83 (0,91)	t (546) = −1,36; *p* = 0,174	−0,15	F (1,538) = 1,78; *p* = 0,183	0,003
i: Suizidgedanken	0,38 (0,63)	0,32 (0,68)	t (546) = 0,81; *p* = 0,421	0,09	F (1,538) = 0,43; *p* = 0,510	0,001
GAD‑2^c^						
a: Nervosität oder Ängstlichkeit	1,83 (0,92)	1,82 (0,94)	t (546) = 0,13; *p* = 0,899	0,01	F (1,538) = 0,20; *p* = 0,651	0,000
b: Gedankenkreisen	1,64 (1,00)	1,60 (0,90)	t (546) = 0,37; *p* = 0,709	0,04	F (1,538) = 0,29; *p* = 0,591	0,001
*SSS‑8*^d^						
1: Bauchschmerzen oder Verdauungsbeschwerden	1,40 (1,23)	1,21 (1,18)	t (546) = 1,45; *p* = 0,148	0,16	F (1,538) = 2,47; *p* = 0,117	0,005
2: Rückenschmerzen	1,73 (1,22)	1,74 (1,23)	t (543) = −0,09; *p* = 0,930	−0,01	F (1,535) = 0,02; *p* = 0,896	0,000
3: Schmerzen in Armen, Beinen oder Gelenken	1,39 (1,29)	1,39 (1,31)	t (545) = 0,05; *p* = 0,957	0,01	F (1,537) = 0,32; *p* = 0,574	0,001
4: Kopfschmerzen	1,46 (1,17)	1,51 (1,14)	t (542) = −0,41; *p* = 0,680	−0,04	F (1,534) = 0,00; *p* = 0,990	0,000
5: Schmerzen im Brustbereich oder Kurzatmigkeit	1,07 (1,18)	0,91 (1,12)	t (542) = 1,28; *p* = 0,200	0,14	F (1,534) = 1,29; *p* = 0,258	0,002
6: Schwindel	0,92 (1,09)	0,94 (1,13)	t (543) = −0,25; *p* = 0,806	−0,03	F (1,535) = 0,03; *p* = 0,860	0,000
7: Müdigkeit	2,84 (1,07)	2,81 (1,03)	t (544) = 0,31; *p* = 0,759	0,03	F (1,536) = 0,47; *p* = 0,496	0,001
8: Schlafstörungen	2,48 (1,32)	2,18 (1,31)	t (544) = 2,07; *p* = 0,975	0,22	F (1,536) = 3,58; *p* = 0,590	0,007
*SOSES*^e^						
1: Gelassenheit	2,69 (1,36)	2,81 (1,37)	t (546) = −0,77; *p* = 0,441	−0,08	F (1,538) = 0,41; *p* = 0,523	0,001
2: Problemlösung	3,14 (1,19)	3,10 (1,17)	t (546) = 0,32; *p* = 0,750	0,03	F (1,538) = 0,25; *p* = 0,617	0,000
3: Zuversicht	2,52 (1,26)	2,76 (1,35)	t (546) = −1,75; *p* = 0,080	−0,19	F (1,538) = 1,87; *p* = 0,172	0,003
4: Sicherheit aufgrund von Erfahrungswerten	3,00 (1,28)	3,07 (1,30)	t (546) = −0,50; *p* = 0,616	−0,05	F (1,538) = 0,27; *p* = 0,603	0,001
5: Zielrealisation	2,75 (1,27)	2,88 (1,33)	t (545) = −0,95; *p* = 0,345	−0,10	F (1,537) = 0,35; *p* = 0,554	0,001
6: Adäquate Anforderungen	3,00 (1,22)	3,02 (1,27)	t (546) = −0,16; *p* = 0,875	−0,02	F (1,538) = 0,00; *p* = 0,990	0,000

Der signifikante Wert des SSS-8: Item 8 *p* < 0,05 im t‑Test wurde anhand der Bonferroni-Holm-Korrektur adjustiert und adjustiert in der Tabelle vermerkt; in der ANCOVA mit Unternehmensgröße als Zwischensubjektfaktor wurde für die Variablen Alter, Geschlecht, Wochenarbeitsstunden, Schichtdienst und Führungsposition kontrolliert

*SD* Standardabweichung; *df* Freiheitsgrade

^a^[26]

^b^[27]

^c^[29]

^d^[31]

^e^[34]

*p < 0,05; **p < 0,01

### Zusammenhang zwischen dem PSC-4 und klinischen Parametern

Korrelationen und interne Konsistenzen sind in Tab. 4 und 5 aufgeführt. Die Schätzung der internen Konsistenzen mithilfe von Cronbachs Alpha zeigte akzeptable bis gute Werte für alle Skalen. Zusammenfassend zeigten sich erwartungskonforme Korrelationen bei GU (Tab. 4) zwischen den Items des PSC‑4 und den klinischen Parametern: Das „Engagement des Managements bei der Stressprävention“ korrelierte schwach negativ mit der somatischen Symptombelastung (r = −0,14; *p* < 0,01) und schwach positiv mit der Arbeitsfähigkeit (r = 0,11; *p* < 0,05). Die Items 2 „Gesundheit als Priorität“, 3 „Kommunikation psychosozialer Themen“ und 4 „Partizipation der Beschäftigten“ korrelierten schwach negativ mit Depressivität (Item 2: r = −0,10; *p* < 0,05, Item 3: r = −0,12; *p* < 0,05, Item 4: r = −0,11; *p* < 0,05), somatischer Symptombelastung (Item 2: r = −0,12; *p* < 0,05, Item 3: r = −0,11; *p* < 0,05, Item 4: r = −0,14; *p* < 0,01) und schwach positiv mit Arbeitsfähigkeit (Item 2: r = 0,12; *p* < 0,05, Item 3: r = 0,10; *p* < 0,05, Item 4: r = 0,11; *p* < 0,05). Die Items „Gesundheit als Priorität“ (r = 0,17; *p* < 0,01) und „Partizipation der Beschäftigten“ (r = 0,17; *p* < 0,01) korrelierten schwach positiv mit der berufsbezogenen Selbstwirksamkeit.

**Tab. 4:** Deskriptive Statistik und Korrelationen der Variablen in Großunternehmen (GU)

	GU (*n* = 439) Mittelwert (SD)	1	2	3	4	5	6	7	8	9	10	11
1. PHQ‑9^a,b^ gesamt	12,94 (5,13)	(0,80)	–	–	–	–	–	–	–	–	–	–
2. GAD‑2^c^ gesamt	3,46 (1,72)	0,59**	(0,75)	–	–	–	–	–	–	–	–	–
3. GAF^d^	66,27 (7,76)	−0,40**	−0,30**	–	–	–	–	–	–	–	–	–
4. SSS‑8^e^ gesamt	13,44 (5,82)	0,60**	0,46**	−0,30**	(0,75)	–	–	–	–	–	–	–
5. VR^f^	3,49 (0,71)	0,37**	0,34**	−0,30**	0,46**	–	–	–	–	–	–	–
6. WAI^g^	5,05 (2,38)	−0,39**	−0,28**	0,30**	−0,25**	−0,27**	–	–	–	–	–	–
7. SOSES^h^ gesamt	3,85 (0,97)	−0,35**	−0,30**	0,19**	−0,17**	−0,22**	0,33**	(0,86)	–	–	–	–
8. PSC‑4^i^ Item 1: Engagement	2,52 (1,08)	−0,09	−0,05	0,06	−0,14**	−0,10*	0,11*	0,09	–	–	–	–
9. PSC‑4^i^ Item 2: Priorität	2,75 (1,15)	−0,10*	−0,09	0,04	−0,12**	−0,09	0,12*	0,17**	0,76**	–	–	–
10. PSC‑4^i^ Item 3: Kommunikation	2,67 (1,20)	−0,12*	0,00	0,05	−0,11*	−0,04	0,10*	0,07	0,54**	0,58**	–	–
11. PSC‑4^i^ Item 4: Partizipation	2,46 (1,10)	−0,11*	−0,06	0,09	−0,14**	−0,06	0,11*	0,17**	0,60**	0,57**	0,50**	–

*N* = 422–439; Cronbachs Alpha in Klammern

*SD* Standardabweichung

^a^[26]

^b^[27]

^c^[29]

^d^[25]

^e^[31]

^f^[32]

^g^[33]

^h^[34]

^i^[35]

*p < 0,05; **p < 0,01

**Tab. 5:** Deskriptive Statistik und Korrelationen der Variablen in Klein(st)- und mittleren Unternehmen (KMU)

	KMU (*n* = 109) Mittelwert (SD)	1	2	3	4	5	6	7	8	9	10	11
1. PHQ‑9^a,b^ gesamt	12,7 (5,03)	(0,79)	–	–	–	–	–	–	–	–	–	–
2. GAD‑2^c^ gesamt	3,41 (1,66)	0,56**	(0,76)	–	–	–	–	–	–	–	–	–
3. GAF^d^	66,08 (6,95)	−0,39**	−0,28**	–	–	–	–	–	–	–	–	–
4. SSS‑8^e^ gesamt	12,77 (5,57)	0,67**	0,49**	−0,27**	(0,71)	–	–	–	–	–	–	–
5. VR^f^	3,44 (0,72)	0,50**	0,29**	−0,29**	0,42**	–	–	–	–	–	–	–
6. WAI^g^	5,13 (2,55)	−0,39**	−0,36**	0,47**	−0,31**	−0,42**	–	–	–	–	–	–
7. SOSES^h^ gesamt	3,94 (1,07)	−0,38**	−0,47**	0,35**	−0,22*	−0,29**	0,45**	(0,91)	–	–	–	–
8. PSC‑4^i^ Item 1: Engagement	2,54 (1,11)	0,08	0,06	−0,08	0,10	0,07	−0,02	0,14	–	–	–	–
9. PSC‑4^i^ Item 2: Priorität	2,81 (1,21)	0,07	0,00	−0,01	0,16	0,06	0,04	0,18	0,79**	–	–	–
10. PSC‑4^i^ Item 3: Kommunikation	2,17 (1,16)	−0,00	−0,03	0,02	0,23*	0,06	0,04	0,12	0,50**	0,62**	–	–
11. PSC‑4^i^ Item 4: Partizipation	2,30 (0,99)	−0,06	−0,09	0,09	0,02	−0,01	0,13	0,16	0,60**	0,53**	0,61**	–

*N* = 106–109; Cronbachs Alpha in Klammern

^a^[26]

^b^[27]

^c^[29]

^d^[25]

^e^[31]

^f^[32]

^g^[33]

^h^[34]

^i^[35]

*p < 0,05; **p < 0,01

In KMU (Tab. 5) hingegen zeigten sich, bis auf schwach positive Korrelationen von „Kommunikation psychosozialer Themen“ des PSC‑4 und somatischer Symptombelastung (r = 0,23, *p* < 0,05), keine signifikanten Korrelationen.

## Diskussion

Es wurde der Zusammenhang zwischen Unternehmensgröße und psychosomatischer Gesundheit von insgesamt 548 Teilnehmenden der Interventionsstudie „friaa“ explorativ untersucht. Nutzende der PT‑A aus GU (*n* = 439) und KMU (*n* = 109) unterschieden sich nicht hinsichtlich ihrer im Selbstbericht und Interview erhobenen psychosomatischen Gesundheit, ihres allgemeinen Funktionsniveaus, ihrer Arbeitsfähigkeit und berufsbezogenen Selbstwirksamkeit. Beschäftigte in GU nahmen eine signifikant verstärkte Kommunikation psychosozialer Themen in ihrem Betrieb im Unterschied zu Beschäftigten in KMU wahr. Dieser Unterschied blieb nach Einbezug von Kovariablen bestehen. Während in KMU ein signifikant positiver Zusammenhang zwischen der Kommunikation psychosozialer Themen und somatischer Symptombelastung vorlag, ergaben sich in GU erwartungskonforme Zusammenhänge, z. B. positive mit der arbeitsbezogenen Selbstwirksamkeit und der Arbeitsfähigkeit und negative mit Depressivität und somatischer Symptombelastung. Die Nutzergruppe der PT‑A zeigte sich in GU und KMU ähnlich stark beansprucht: Darauf wiesen selbstberichtete depressive, ängstliche und somatoforme Symptome bei einer Arbeitsfähigkeit im mittleren Bereich hin.

Der Arbeitsplatz als wichtige Stütze im Alltag bietet sich als Zugang für die Prävention psychischer Erkrankungen an [6]. Bisherige Studien untersuchten die Implementierung der PT‑A in GU und stellten ein geringeres Beanspruchungserleben der Nutzenden im Unterschied zur ambulanten Regelversorgung fest [7, 8, 21]. Aufgrund etablierter BGM-Strukturen und daher weniger stigmatisierender Barrieren war zu vermuten, dass in GU die PT‑A zu einem früheren Zeitpunkt einer psychischen Erkrankung in Anspruch genommen werden würde als in KMU. Dies ließ sich aufgrund des ähnlich hohen Beanspruchungserlebens der Beschäftigten aus GU und KMU nicht bestätigen. Hingegen wurde eine stärkere Beeinträchtigung durch Schlafstörungen in GU im Vergleich zu KMU festgestellt. Dieser Unterschied, ähnlich zur in einer Querschnittserhebung im UK gezeigten geringeren Stressbelastung von Beschäftigten in KMU, bestand nach der Kontrolle für Kovariablen nicht mehr [12]. Ob dies durch die Unterschiede in der Altersstruktur, durch multiple Testungen oder durch unterschiedliche Unternehmensstrukturen bedingt war, sollte mit einem expliziten Messinstrument zur Erfassung von Schlafstörungen überprüft werden. Das Ergebnis, dass in GU aus Sicht der Beschäftigten psychosoziale Themen häufiger angesprochen werden als in KMU, hat nicht zu einer früheren Vorstellung und damit zu einer antizipierten geringeren Symptombelastung der Nutzergruppe in GU beigetragen, obwohl die Thematisierung für eine Entstigmatisierung psychischer Störungen relevant ist [38]. So würde die PT‑A eher in Anspruch genommen werden bei weniger wahrgenommenen stigmatisierenden Barrieren [39]. In KMU erfolgte der Zugang zur PT‑A überwiegend durch Eigeninitiative, wohingegen in GU auch der Sozialdienst und BÄD eine wichtige Rolle einnahmen. Dieses Ergebnis steht im Einklang mit dem Befund der häufigeren Thematisierung psychosozialer Themen in GU und verdeutlicht deren etabliertere BGM-Strukturen. Daher sollte v. a. in KMU die Relevanz der Thematisierung psychosozialer Themen, bspw. durch Führungskräfteschulungen, nähergebracht werden. Zu diskutieren ist, ob während der Corona-Zeit digitale Kommunikationsangebote in KMU schlechter ausgebaut waren als in GU, sodass psychosoziale Themen weniger kommuniziert wurden.

Wie das ähnlich hohe Beanspruchungserleben der Beschäftigten in GU und KMU zeigte, könnten sowohl die bessere Implementierung der Gefährdungsbeurteilung psychischer Belastung [13] und eine Ableitung von verhältnispräventiven Maßnahmen als auch verhaltenspräventive Ansatzpunkte, wie bspw. die weitere Etablierung der PT‑A, hilfreich sein [40], um die psychische Gesundheit zu unterstützen und die Arbeitskraft zu wahren [41]. Zum Beispiel erreichte die PT‑A auch 14 % der Teilnehmenden aus GU und 20 % aus KMU mit Funktionseinschränkung ohne psychische und Verhaltensstörung und daher in einem Frühstadium. Unabhängig von der Unternehmensgröße konnten Faktoren wie explizite Richtlinien und staatliche Zuschüsse [22] identifiziert werden, die es Unternehmen erleichtern, gesundheitsfördernde Programme zu etablieren. Bisherige Forschung zeigte, dass kleinere Unternehmen weniger häufig Gesundheitsinterventionen anbieten [18, 22], obwohl Beschäftigte in KMU und GU ein ähnliches Interesse daran haben [39]. Aufgrund des finanziellen und personellen Ressourcenmangels können KMU diesen Wünschen oft nicht nachkommen [18, 42]. Deshalb wurden erste Programme etabliert, um gezielt die Stressbewältigung von Führungskräften in KMU zu fördern, wie bspw. im Rahmen des Projektes „KMU GO!“ [43]. Die Ergebnisse eines systematischen Reviews [44] deuten darauf hin, dass kognitiv-behaviorale Interventionen bei depressiven und ängstlichen Symptomen von Beschäftigten in KMU wirksam sind.

So sollte das PSC verbessert werden, da es im positiven Zusammenhang mit Arbeitsbedingungen, Gesundheit und Engagement der Beschäftigten steht [17, 35, 45], was sich auch in dieser Studie für Beschäftigte v. a. aus GU bestätigen ließ. Auch die positiven Zusammenhänge des PSC mit der arbeitsbezogenen Selbstwirksamkeit der Beschäftigten aus KMU unterstützen die Relevanz des PSC. Das PSC ist also ein wichtiger Ansatzpunkt für verhältnispräventive Interventionen in Unternehmen. Im Rahmen dieser Studie steht in KMU, anders als in GU, die vermehrte Kommunikation psychosozialer Themen in einem schwach positiven Zusammenhang mit somatischer Symptombelastung. Es ist zu diskutieren, dass aufgrund der Arbeitsbedingungen in KMU wie in Handwerksbetrieben die physische Gesundheit einen hohen Stellenwert besitzt und somit psychosoziale Aspekte öfter thematisiert werden müssen. Anders als in GU tragen möglicherweise in KMU, in denen das Thema psychische Gesundheit weniger präsent ist, nicht das PSC, sondern eher flache Hierarchien mit kurzen Kommunikationswegen und guten zwischenmenschlichen Beziehungen als protektive Faktoren zur Gesundheit bei. Im Rahmen einer Querschnittserhebung aus dem UK wirkte sich die Unternehmensgröße nicht direkt, sondern über den vermittelnden Effekt der Arbeitsbedingungen auf die psychische Beanspruchung der Beschäftigten aus [19]. Insbesondere quantitative Arbeitsüberlastung [12], Arbeitsplatzunsicherheit, schlechtere Karrieremöglichkeiten, mangelnde Kommunikation und gute Arbeitsbeziehungen stehen stärker im Zusammenhang mit dem Stresserleben von Beschäftigten in KMU. In GU scheinen qualitative Arbeitsüberlastung, geringere Autonomie am Arbeitsplatz und die geringe Einbindung von Mitarbeitenden entscheidender zu sein [12]. Außerdem wurde festgestellt [46], dass der Berufssektor in der Beurteilung psychischer Beanspruchung wichtiger ist als die Unternehmensgröße. So sollten neben der Unternehmensgröße weitere Variablen wie Branche, Umsatz und Arbeitsplatz berücksichtigt werden [46, 47]. Auch eine größere Fallzahl mit einer repräsentativen Aufteilung in Kleinst-, kleine, mittlere und Großunternehmen und einer Replikation außerhalb der Corona-Zeit könnten neue Erkenntnisse erbringen.

Limitierend sind die explorative Natur sowie multiple Testungen, weswegen die Ergebnisse als vorläufig zu betrachten sind. Für eine bessere Vergleichbarkeit der Gruppen sollte besonderer Wert auf die Rekrutierung von Teilnehmenden aus KMU gelegt werden [44]. Auch die Interpretation der Korrelationskoeffizienten liefert erste Hinweise, sollte aufgrund der unterschiedlich großen Gruppen in GU und KMU mit einem umfangreicheren Messinstrument des PSC überprüft werden. Die Betriebszugehörigkeit und damit auch die Reichweite der PT‑A wurden aus Gründen der Anonymität nicht berücksichtigt. Es handelte sich um eine selektive Stichprobe von Beschäftigten, die Interesse an der Inanspruchnahme einer PT‑A hatte. Der Vergleich zwischen GU und KMU sollte in Abhängigkeit von unterschiedlichen Kontexten untersucht werden. Die Fallzahl der vorliegenden Stichprobe mit 548 Teilnehmenden und die bundesweiten Rekrutierungen von Beschäftigten über unterschiedliche Betriebe und Kanäle spricht für eine hohe externale Validität.

Zusammenfassend liefern die Daten erste Hinweise, dass psychosoziale Themen in GU aus Sicht der Beschäftigten häufiger thematisiert werden als in KMU, was als wichtige Ressource in GU gefördert und in KMU verbessert werden sollte. Bezüglich der psychosomatischen Gesundheit scheint es keine Unterschiede in den Nutzergruppen aus GU und KMU trotz unterschiedlicher Strukturen zu geben und ähnlich stark beanspruchte Beschäftigte wurden mittels einer PT‑A erreicht. Anlässlich des hohen Beanspruchungserlebens sollte die Bereitschaft des Managements, Maßnahmen für die psychische Gesundheit der Beschäftigten zu treffen, sowohl in GU als auch in KMU, verbessert werden und dabei die unternehmensspezifischen Ressourcen genutzt werden. Eine Ressource, die bei Beschäftigten aus GU und KMU vorlag, ist die leicht positive Selbstwirksamkeit. Eine hohe Selbstwirksamkeit kann sich positiv auf die psychische Gesundheit auswirken [48] und sollte im Rahmen von verhaltenspräventiven Angeboten wie der PT‑A gestärkt werden. Auch verhältnispräventive Maßnahmen zur Förderung des psychischen Wohlbefindens von Beschäftigten sollten sowohl auf betrieblicher als auch auf politischer Ebene getroffen werden.

## Footnotes

1 https://www.destatis.de/DE/Themen/Branchen-Unternehmen/Unternehmen/Kleine-Unternehmen-Mittlere-Unternehmen/aktuell-beschaeftigte.html. Zugegriffen: 02.01.2024.

2 https://www.destatis.de/DE/Themen/Branchen-Unternehmen/Unternehmen/Kleine-Unternehmen-Mittlere-Unternehmen/Glossar/kmu.html#:~:text=Der%20Begriff%20KMU%20umfasst%20Kleinst%C2%ADunternehmen%2C%20kleine%20Unternehmen%20und,KMU%20umfasst%20Kleinst%C2%ADunternehmen%2C%20kleine%20Unternehmen%20und%20mittlere%20Unternehmen. Zugegriffen: 22.10.2023.
